# An exploratory simulation of the optimization of leukocyte transmigration, indicating that barrier routing is governed by context-dependent biochemical cues rather than hardwired programs

**DOI:** 10.3389/fimmu.2026.1863910

**Published:** 2026-10-02

**Authors:** Piotr Religa, Michel-Edwar Mickael, Norwin Kubick, Jarosław Olav Horbańczuk, Mariusz Sacharczuk

**Affiliations:** 1 Department of Medicine, Karolinska Institute, Solna, Sweden; 2 Institute of Genetics and Animal Biotechnology, Polish Academy of Sciences, Jastrzebiec, Poland; 3 Department of Biology, Institute of Plant Science and Microbiology, University of Hamburg, Hamburg, Germany

**Keywords:** mathematical modeling, paracellular and transcellular transport, T cell, Th1, Th17

## Abstract

**Introduction:**

Leukocyte infiltration across the blood-brain barrier contributes to many neuroinflammatory disorders, yet the physical mechanisms that determine how immune cells cross the barrier remain incompletely understood. In particular, it is unclear how leukocytes choose between paracellular migration through endothelial junctions and transcellular migration through the endothelial cell body. This question is especially relevant during central nervous system inflammation, where different T-lymphocyte subsets, including Th1 and Th17 cells, appear to preferentially use distinct migration routes. Previous studies have examined individual mechanical forces or upstream biochemical signaling pathways, but these approaches have not provided a unified framework that considers the energetic contributions of both the leukocyte and the deforming endothelium.

**Methods:**

To address this, we developed a two-route energy minimization model that incorporates an anisotropic, fiber-reinforced hyperelastic model, junctional unzipping, Helfrich membrane bending, caveolar vesicle fusion, and cytokine-dependent signaling. Model parameters were optimized, and route switching was evaluated using Sobol global sensitivity analysis to determine which specific mechanical and signaling parameters independently drive route switching rather than merely scaling overall energy magnitude.

**Results:**

The model reproduced the experimental validation conditions used in this study and identified distinct regimes of mechanical control. Under IFN-γ-dominant conditions, route selection was influenced primarily by ICAM-1 adhesion kinetics. Under IL-17-dominant conditions, control shifted toward PECAM-1-related physical properties and their nonlinear interactions, which accounted for 76% of the variance in route switching.

**Discussion:**

These exploratory findings suggest that leukocyte transmigration is shaped by local biochemical conditions together with the mechanical properties of the endothelial barrier, rather than by a single fixed migration program. Mapping the associated energetic landscape may help clarify the physical factors governing immune-cell entry into the central nervous system and provide a framework for studying mechanisms that regulate leukocyte trafficking during neuroinflammation.

## Graphical Abstract

**Figure:**
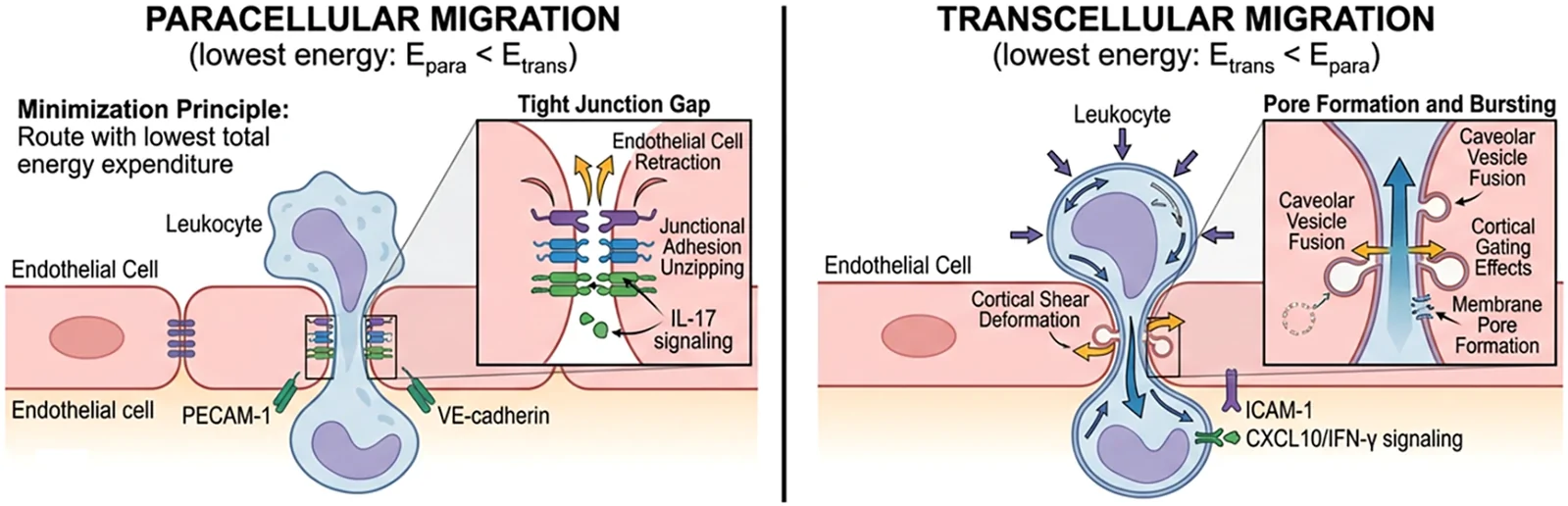

## 1 Introduction

The blood-brain barrier is maintained by a specialized cerebrovascular endothelium characterized by tight junctions, minimal transcytosis, and restricted paracellular permeability. In multiple sclerosis and other neuroinflammatory conditions, circulating T lymphocytes breach this barrier to gain access to the central nervous system parenchyma, a process that contributes directly to lesion formation and neurological dysfunction (1, 2). Two anatomically and mechanistically distinct routes of transmigration have been documented at the blood-brain barrier: paracellular transit through the junction between adjacent endothelial cells, and transcellular transit through the endothelial cell body via caveolae-mediated membrane channels or vacuolar organelles (3, 4). The two routes have different consequences for barrier integrity: paracellular transit involves transient junction opening and is associated with secondary increases in paracellular permeability, while transcellular transit preserves junction continuity and may therefore represent a more contained mode of immune cell passage (5, 6). Understanding the determinants of route selection thus has both mechanistic and potential therapeutic relevance in neuroinflammatory disease.

The central biophysical problem is that the same T lymphocyte, interacting with the same endothelial monolayer, selects different transmigration routes depending on the cytokine environment, yet neither cell appears to make a predetermined route decision. Experimental studies have established cytokine-route associations: IFN-γ-producing Th1 cells preferentially use the transcellular route at the blood-brain barrier, while IL-17-producing Th17 cells preferentially use the paracellular route (7). However, the physical mechanism by which these cytokine states translate into route decisions has not been formally described. Route selection likely reflects a balance between the mechanical cost incurred by the leukocyte in each route and the mechanical cost incurred by the endothelial cell in yielding along each route, with the route of least total cost emerging from the concurrent responses of both cells. This framing — route selection as an emergent outcome of two simultaneous energy minimization processes — has not been previously formulated or tested computationally.

Previous computational studies of leukocyte transmigration have addressed individual components of the process. Contact mechanics and membrane deformation models have characterized the forces required for leukocyte squeezing through narrow gaps (8). The role of ICAM-1 clustering in initiating transcellular transit and the redirection of Th1 cells to paracellular routes following Cav-1 knockdown have been documented experimentally (4, 6). The demonstration that IL-17-driven RhoA/ROCK signaling loosens endothelial junctions and that this is associated with paracellular Th17 transmigration provided a cytokine-mechanical link (7, 9). However, these studies characterized individual routes in isolation or attributed route preference to upstream signaling without a unified energy framework that accounts for both cellular contributions simultaneously. In particular, no prior model has identified which parameters are capable of independently driving route switching as distinct from parameters that modulate energy magnitude without changing the route decision.

In this study, we develop a two-route energy minimization model of leukocyte transmigration at the blood‐brain barrier and apply it to characterize the switching manifold separating paracellular and transcellular routes. The paracellular energy equation combines Holzapfel-Ogden hyperelastic constitutive mechanics (10) for both cells with a cooperative junction unzipping cascade and a Brownian ratchet actin polymerization term (11). The transcellular energy equation integrates Neo-Hookean leukocyte compression, Helfrich membrane bending mechanics, leukocyte cortical pressure as an active mechanical lever, a caveolae sequential-burst fusion chain informed by Cav-1 membrane reservoir thermodynamics, and active ICAM-1 ligation and clustering kinetics. The model is optimized using the gradient-based L-BFGS-B algorithm within a randomized multi-start framework to ensure parameter identifiability, and route-switching drivers are isolated using Sobol global sensitivity analysis applied to the switching energy difference (12, 13). Finally, the continuous cytokine parameter manifold is mapped using Brent root-finding and validated against held-out experimental knockout conditions.

## 2 Methods

### 2.1 Model overview

Leukocyte transmigration across the endothelium is modeled using a thermodynamic energy-minimization framework. The cell chooses between two distinct physical pathways: paracellular (passing between endothelial cells through disrupted junctions) and transcellular (forming a membrane tunnel directly through an individual endothelial cell). For a given cellular phenotype and cytokine condition, the total energy barriers for paracellular migration (*E*_parac_) and transcellular migration (*E*_trans_) are calculated. The pathway energy gap is defined as:

(1) $$\text{Δ}E=E_{\text{trans}}-E_{\text{parac}}$$

A negative energy gap ( $\text{Δ}E<-E_{\text{margin}}$) favors transcellular transmigration, whereas a positive gap ( $\text{Δ}E>+E_{\text{margin}}$) favors paracellular transmigration, where $E_{\text{margin}}=500\text{ aJ}$ represents the biological energy threshold accounting for cell-to-cell variability.

#### 2.1.1 Dimensionality of concentration notations:

Throughout the manuscript, square brackets [ … ] denote volumetric (3D) concentrations in the extracellular space or bulk cytosol, expressed in pg/mL or M (e.g., [IFN-γ], [IL-17], [CXCL10], and conc_A). In contrast, cell surface densities are designated by ρ and represent 2D area-bound molecular concentrations on the plasma membrane expressed in m^-^² (e.g., ρ_ICAM, ρ_PECAM1,2D, ρ_VE-cadherin). Dimensionless ratio parameters (such as β_cav,eff) represent local relative kinetic enrichment factors rather than spatial concentrations.

### 2.2 Energy equations

Paracellular Route Mechanics (*E*_parac_) The total paracellular barrier energy combines leukocyte deformation, endothelial cell retraction, junctional unzipping, actin polymerization drive, and active cytokine-mediated machinery:

(2) $$E_{\text{parac}}=E_{\text{L,deform}}+E_{\text{EC,retract}}+E_{\text{unzip}}-E_{\text{poly}}+E_{\text{active}}$$

Leukocyte Deformation (*E*_L,deform_): Modeled using a strain-energy density formulation based on the Holzapfel–Ogden hyperelastic material model over the cortical volume *V*_cortex_:

The Holzapfel–Ogden framework models hyperelastic cell deformation by splitting the energy into an isotropic ground-matrix response (cytosolic volume resistance) and an anisotropic component driven by fiber stretching along primary structural axes. This captures how directional actin stress fiber tension actively resists squeezing as the leukocyte forces its way into narrow endothelial junctions.

(3) $$\lambda_{1}=\frac{g_{\text{eff}}}{2R_{\text{L}}}$$

where *ɡ*_eff_ is the effective junctional gap, and *R*_L_ is the leukocyte radius. Cortical stiffening induced by CXCL10 is incorporated via receptor occupancy:

(4) $$\phi_{CXCL10}=\frac{\left[CXCL10\right]}{K_{CXCL10}+\left[CXCL10\right]}$$

(5) $$E_{\text{L,deform}}=E_{\text{HO}}\left(\lambda_{1}\right)\cdot V_{\text{cortex}}\cdot \left(1+\left(s_{\text{max}}-1\right)\cdot \phi_{CXCL10}\cdot 1_{\text{Δ}G_{CXCL10}>0}\right)$$

Endothelial Retraction (*E*_EC,retract_): Calculates the hyperelastic strain energy of endothelial cell retraction required to open the junction:

(6) $$\lambda_{\text{EC}}=1+\frac{g_{\text{eff}}}{2W_{\text{EC}}}$$

(7) $$E_{\text{EC,retract}}=E_{\text{HO}}\left(\lambda_{\text{EC}}\right)\cdot V_{\text{EC,ref}}$$

Junction Unzipping (*E*_unzip_): Sequential bond rupture energy across junctional adhesion molecule cascades (PECAM-1, VE-cadherin, claudin, occludin, JAMs):

(8) $$E_{\text{unzip}}=\sum_{i}\rho_{i,\text{eff}}\cdot \in_{i}\cdot L_{\text{junc}}\cdot P_{i}$$

where *P_i_* provides a continuous penalty transition based on drive forces relative to rupture thresholds ( $F_{\text{thr},i}=\frac{\rho_{i}\in_{i}L_{\text{junc}}}{\xi_{i}}$):

(9) $$P_{i}=\text{smooth\_max}\left(1.0,\frac{F_{\text{thr},i}}{F_{\text{drive}}},\in_{\text{smooth}}\right)$$

PECAM-1 density is modulated by IL-17 using a non-saturating inverse-sensitivity factor:

(10) $$\rho_{PECAM1,eff}=\frac{\rho_{PECAM1,2D}}{1+\beta_{IL17}\cdot \left[IL-17\right]}$$

Actin Polymerization Drive (*E*_poly_): Entropic force generation at the pseudopod tip (*A*_tip_) driven by monomeric actin (*N*_fil_ filaments):

(11) $$E_{\text{poly}}=N_{\text{fil}}k_{\text{B}}T\ln\left(1+\frac{k_{\text{on}}C_{A}\delta a}{k_{\text{off}}\alpha_{\text{store}}}\right)$$

Active IL-17 Signaling (*E*_active_): Energetic reduction supplied by IL-17 active signaling:

(12) $$E_{\text{active}}=-\text{Δ}G_{IL17}\left(\frac{\left[IL-17\right]}{K_{IL17}+\left[IL-17\right]}\right)$$

Transcellular Route Mechanics (*E*_trans_): The total transcellular barrier energy integrates cytosolic deformation, cortical displacement, caveolar membrane remodeling, vesicle fusion, burst mechanics, chemokinetag guidance, ICAM-1 ligation, and cortical gating:

(13) $$E_{\text{trans}}=E_{\text{Lc}}+E_{\text{cortex,L}}+E_{\text{mem}}+E_{\text{fus}}+E_{\text{burst}}-E_{\text{poly,trans}}+E_{CXCL10}+E_{\text{ICAM}}+E_{\text{gate}}$$

Cytosolic Compression (*E*_Lc_): Bulk hyperelastic strain energy integrated over the compressed cytosolic volume *V*_cyt_:

(14) $$E_{\text{Lc}}=\int_{V_{\text{cyt}}}(\frac{\mu_{\text{cyto}}}{2}\left(I_{1}-3\right)+\frac{K_{\text{cyto}}}{2}\left(J-1{)}^{2}\right)dV$$

where C = F^T^F is the right Cauchy–Green deformation tensor measuring strain independent of rigid-body rotations, in which F = ∇_X is the deformation gradient mapping initial material coordinates X to spatial coordinates x, where *I*_1_ = tr(**C**) is the first invariant of the right Cauchy–Green deformation tensor **C**, *J* = *det*(**F**) is the volume ratio, and *μ*_cyto_ and *K*_cyto_ are the cytosolic shear and bulk moduli.

Cortical Shear Strain (*E*_cortex,L_): Area-integrated elastic energy penalty from local cortical stretch and membrane-skeleton area dilation around the indentation zone (*A*_pore_):

(15) $$E_{\text{cortex,L}}=\int_{A_{\text{pore}}}\left(\gamma_{\text{cortical}}\cdot \frac{\text{Δ}A}{A_{0}}+\frac{\mu_{\text{cortex}}}{2}\left(\lambda_{\theta }^{2}+\lambda_{\theta }^{-2}-2\right)\right)dA$$

where *γ*_cortical_ is baseline cortical tension, Δ*A*/*A*_0_ is relative area dilation, *μ*_cortex_ is the 2D cortical shear modulus, and *λ_θ_* is the circumferential stretch ratio around the pore radius *R*_pore_.

Caveolar Remodeling & Tension Mitigation (*E*_mem_): Membrane energy associated with forming the transcellular pore (*R*_t_) and lining it with effective caveolar units (*n*_cav,eff_):

(16) $$E_{\text{mem}}=\left(n_{\text{cav,eff}}\cdot 2\pi \kappa_{\text{b}}\right)+\left(\gamma_{\text{eff}}\cdot 2\pi R_{\text{t}}d_{\text{EC}}\right)$$

Vesicle Fusion Energy (*E*_fus_): Free energy required to incorporate caveolar vesicles into the developing pore:

(17) $$E_{\text{fus}}=n_{\text{cav,eff}}\cdot \text{Δ}G_{\text{fuse}}$$

Pore Nucleation & Burst Energy (*E*_burst_): Work required to break bilayer continuity against line tension (Γ_line_):

(18) $$E_{\text{burst}}=2\pi R_{\text{pore}}{\text{Γ}}_{\text{line}}-\pi R_{\text{pore}}^{2}\gamma_{\text{eff}}$$

Transcellular Actin Polymerization (*E*_poly,trans_): Forward thermodynamic propulsion supplied by polymerized actin filaments (*N*_fil,trans_):

(19) $$E_{\text{poly,trans}}=N_{\text{fil,trans}}k_{\text{B}}T\text{ln}\left(1+\frac{k_{\text{on}}C_{A}\delta a}{k_{\text{off}}\alpha_{\text{store}}}\right)$$

CXCL10 Guidance Energy (*E*_CXCL10_): Energetic bias resulting from CXCL10 receptor activation:

(20) $$\phi_{CXCL10}=\frac{\left[CXCL10\right]}{K_{CXCL10}+\left[CXCL10\right]}$$

(21) $$E_{CXCL10}=-\text{Δ}G_{CXCL10}\cdot \phi_{CXCL10}$$

Cytokine & ICAM-1 Coupling Machinery: ICAM-1 ligation drives caveolar recruitment and tension relief (γ_eff_):

(22) $$\phi_{\text{ICAM}}=\frac{\rho_{\text{ICAM}}\cdot \in_{\text{ICAM}}}{K_{\text{ICAM}}+\rho_{\text{ICAM}}\cdot \in_{\text{ICAM}}}$$

(23) $$\beta_{\text{cav,eff}}=1+\left(\beta_{\text{cav}}-1\right)\cdot \phi_{\text{ICAM}}$$

(24) $$\gamma_{\text{eff}}=\frac{\gamma_{\text{mem}}}{\beta_{\text{cav,eff}}}$$

(25) $$f_{\text{cluster}}=\frac{\beta_{\text{cav,eff}}-1}{\beta_{\text{cav,max}}-1}$$

IFN-γ-CAV1 interaction: stimulation scales caveolar reserve capacity using a hyperbolic transition:

(26) $${\text{fold}}_{Cav1}=1+\left({\text{fold}}_{\text{cap}}-1\right)\cdot \frac{\alpha_{\text{IFN}\gamma }\left[\text{IFN-}\gamma \right]}{1+\alpha_{\text{IFN}\gamma }\left[\text{IFN-}\gamma \right]}$$

ICAM-1 Adhesion Energy (*E*_ICAM_):

(27) $$E_{\text{ICAM}}=-\rho_{\text{ICAM,eff}}\cdot \in_{\text{ICAM}}\cdot A_{\text{contact}}\cdot f_{\text{cluster}}$$

Cortical Gating Penalty (*E*_gate_): Energy penalty imposed when effective cortical pressure exceeds cortical tension capacity:

(28) $$E_{\text{gate}}=max\left(0,\frac{2\gamma_{\text{eff}}}{R_{\text{c}}}-\sigma_{\text{cortex,L}}\right)A_{\text{contact}}h_{\text{mem}}$$

### 2.3 Parameterization & optimization protocol

Parameter Normalization: Optimization parameters are bounded across multiple orders of magnitude (10^−21^ to 10^15^). To eliminate scale disparities during gradient calculations, parameters spanning more than two decades are mapped logarithmically into unit space u∈[0,1]; narrow-range parameters are mapped linearly:

(29) $$u_{i}=\{\begin{array}{cc} \frac{\text{ln}\left(t_{i}/{\text{LO}}_{i}\right)}{\text{ln}\left({\text{HI}}_{i}/{\text{LO}}_{i}\right)}, & \text{if }\log_{10}\left({\text{HI}}_{i}/{\text{LO}}_{i}\right)>2.0\\ \frac{t_{i}-{\text{LO}}_{i}}{{\text{HI}}_{i}-{\text{LO}}_{i}}, & \text{otherwise} \end{array}$$

Loss Function & Multi-Start Protocol: Model optimization uses a smooth, margin-maximizing exponential loss function over training conditions:

(30) $$ℒ\left(u\right)=\sum_{c\in \text{Conditions}}\exp{\left(-\text{clip}\left(\frac{m_{c}}{\text{SOFTNESS}},-50,3.0\right)\right)}^{2}$$

where $m_{c}=\frac{\text{Δ}E_{c}-E_{\text{margin}}}{E_{\text{margin}}}>0$ for paracellular preferences and $m_{c}=\frac{-\text{Δ}E_{c}-E_{\text{margin}}}{E_{\text{margin}}}$ for transcellular preferences (SOFTNESS = 50.0).

Optimization is performed using L-BFGS-B (and validated with Trust Region Reflective, TRF). To avoid single-point solution bias within underdetermined parameter spaces, a multi-start validation protocol (*N* = 50) random starts in normalized unit space) evaluates model stability and parameter identifiability.

Validation Strategy (Train/Validate Split): Model parameters were fitted to three training conditions:

- Th1 (Transcellular preferred).
- Th17 (Paracellular preferred).
- Th1 ICAM1-KO (Paracellular preferred.

Model predictions are evaluated against held-out validation conditions without re-fitting.

- Th1: Cav1-KO.
- Th1: CXCL10-KO.
- Th1: CXCL10-KO/Cav1-KOA.

The prediction was classified as structural if 100% of feasible multi-start solutions correctly predict the target route preference.

### 2.4 Parameter sensitivity.

Bounded Route Preference Formulation: To map unbounded energetic differences ( $\text{Δ}E=E_{\text{trans}}-E_{\text{parac}}$, expressed in Joules) onto a normalized, population-interpretable scale across continuous cytokine gradients, the pathway energy gap is transformed using a hyperbolic tangent mapping:

(31) $$s=\tanh\left(\frac{\text{Δ}E}{E_{\text{scale}}}\right)$$

where s∈[−1,+1] represents the directional pathway preference (*s* = −1 corresponds to fully transcellular transmigration, *s* = 0 indicates thermodynamic parity, and *s* = +1 corresponds to fully paracellular transmigration).The scaling factor *E*_scale_ represents the biophysical cell-to-cell variability in the pathway energy gap Δ*E*. To ground *E*_scale_ in reference mechanics, it is defined as 20% of the minimum paracellular energy barrier evaluated across all experimental baseline conditions:

(32) $$E_{\text{scale}}=0.20\times \underset{c\in \text{Conditions}}{min}\left(E_{\text{parac},c}\left(\theta^{*}\right)\right)$$

where θ^*^ denotes the optimal parameter vector. Assuming a unimodal distribution of individual cell energy barriers with variance governed by *E*_scale_, the predicted macroscopic population percentage taking the transcellular route (*P*_trans_) is derived from *s* as:

(33) $$P_{\text{trans}}=100\times \left(\frac{1-s}{2}\right)$$

Cytokine Dose-Response Analysis: To quantify pathway switching along continuous biochemical axes without altering baseline structural parameters, cytokine dose-response sweeps are evaluated while holding cell-intrinsic mechanics constant at optimized parameter sets $\theta \in \Theta_{\text{feasible}}$ For the IL-17 dose-response evaluation, the background cytokine concentrations are fixed at baseline Th1 levels ( IFN-γ=1000 pg/mL, CXCL10=500 pg/mL), and IL-17 concentration is logarithmically sampled across a 4-decade range ( $\text{[IL-}17]\in \left[1,10^{4}\right]\text{pg/mL}$):

(34) $$\text{IL-}17_{\text{grid}}=\{{\left[\text{IL-}17\right]}_{k}|\log_{10}{\left[\text{IL-}17\right]}_{k}\in \left[0,4\right],\;k=1,\ldots ,120\}$$

For each parameter solution *θ_m_* in the multi-start feasible pool Θ_feasible_ (*m* = 1, …, *M*), the critical switching concentration ${\left[IL-17\right]}_{\text{switch},m}$ where the two routes become thermodynamically equivalent (Δ*E* = 0, or *s* = 0) is determined by root-finding via Brent’s method:

(35) $$\text{Δ}E\left(\theta_{m},\text{IFN-}\gamma =1000,{\left[\text{IL-}17\right]}_{\text{switch},m},\text{CXCL}10=500\right)=0$$

The uncertainty band across all feasible fits defines the structural stability interval for the cytokine-induced route switch point.3. Two-Dimensional Cytokine Phase Space Mapping, To evaluate route selection across heterogeneous inflamed tissue microenvironments, the model evaluates pathway preferences over a two-dimensional concentration domain spanning IFN-γ∈[1,3000]pg/mL and IL-17∈[1,3000]pg/mL evaluated on a 40×40 logarithmic grid. Because CXCL10 expression is downstream of IFN-γ signaling, its concentration is dynamically coupled to IFN-γ along the experimental trajectory connecting reference Th1 and Th17 phenotypes:

(36) $$\text{[CXCL}10]\left(\text{IFN-}\gamma \right)=\text{clip}\left(50.0+0.4545\times \left(\text{IFN-}\gamma -10.0\right),\;0,\;10^{4}\right)$$

Alternatively, CXCL10 can be fixed at an intermediate constant background ( [CXCL10]=275 pg/mL). For each grid point $\left(\text{IFN-}\gamma_{i},\text{IL-}17_{j}\right)$, two surface outputs are calculated: Nominal Route Preference Surface (*s*_best_): Evaluated using the primary parameter set θ^*^:

(37) $$s_{\text{best}}\left(i,j\right)=\tanh\left(\frac{\text{Δ}E\left(\theta^{*},\text{IFN-}\gamma_{i},\text{IL-}17_{j},\text{[CXCL}10]\left(\text{IFN-}\gamma_{i}\right)\right)}{E_{\text{scale}}}\right)$$

Structural Agreement Metric (*A_ij_*): Measures the voting consensus across all *M* solutions in the multi-start parameter ensemble **Θ**_feasible_:

(38) $$v_{\text{trans}}\left(i,j\right)=\sum_{m=1}^{M}1_{\left\{s\left(\theta_{m},\text{IFN-}\gamma_{i},\text{IL-}17_{j},\text{[CXCL}10]\left(\text{IFN-}\gamma_{i}\right)\right)<0\right\}}$$

(39) $$f_{\text{trans}}\left(i,j\right)=\frac{v_{\text{trans}}\left(i,j\right)}{M}$$

(40) $$A_{ij}=max\left(f_{\text{trans}}\left(i,j\right),1-f_{\text{trans}}\left(i,j\right)\right)$$

A location in cytokine space is classified as structurally invariant if *A_ij_* = 1.0 (100% consensus across all feasible parameter sets), whereas *A_ij_* ≈ 0.5 designates regions where route preference depends on underdetermined parameter combinations.

Global Variance-Based Sensitivity Analysis (Sobol Sensitivity Protocols): To quantify the sensitivity of the pathway energy gap ( $\text{Δ}E=E_{\text{trans}}-E_{\text{parac}}$, expressed in attojoules) to parameter variations and to resolve parameter interactions across distinct biochemical environments, a global variance-based Sobol sensitivity analysis was performed.

Parameter Sampling Space and Scaling Transformation: The analysis evaluates sensitivity across *D* parameter dimensions, comprising the 16 free parameters optimized during model calibration (or optionally incorporating key structural mechanical parameters held at physiological bounds). To accommodate parameters spanning multiple orders of magnitude, parameters whose lower (LO*_i_*) and upper (HI*_i_*) bounds span more than two log-decades ( ${\text{log}}_{10}\left({\text{HI}}_{i}/{\text{LO}}_{i}\right)>2.0$) are sampled logarithmically:

(41) $$x_{i}={\text{log}}_{10}\left(\theta_{i}\right),\;{\text{for log}}_{10}\left(\frac{{\text{HI}}_{i}}{{\text{LO}}_{i}}\right)>2.0$$

Parameters spanning narrow bounds are sampled on a linear scale.

Quasi-Monte Carlo simulation: space-filling matrices are generated using Saltelli’s sampling scheme with a base sample size of *N*_base_ = 1024. For second-order interaction calculations (SECOND_ORDER = True), the total number of model evaluations per cytokine context is $N_{\text{eval}}=N_{\text{base}}\times \left(2D+2\right)$. Unsampled baseline parameters are fixed at the optimal L-BFGS-B parameter vector θ^*^.

Inflammatory Cytokine Contexts: Sensitivity metrics are evaluated across four distinct microenvironmental contexts to isolate context-dependent driver parameters:

Th1 Context: IFN-γ=500 pg/mL,   IL-17=0 pg/mLTh17 Context: IFN-γ=0 pg/mL,   IL-17=500 pg/mLMixed Context: IFN-γ=250 pg/mL, IL-17=250 pg/mLPathogenic Th17 Context: IFN-γ=500 pg/mL,  IL-17=500 pg/mLIn each context, the CXCL10 chemokine concentration is dynamically linked to IFN-γ induction ( [CXCL10]=1.0×[IFN-γ] pg/mL).

Signal Conditioning, Outlier Screening, and Quality Control: To prevent numerical instability or non-convergent solutions from corrupting variance calculations, output evaluation arrays undergo strict conditioning: Imputation: Evaluation failures (NaN) are imputed using the context-specific median value of finite evaluations. If the failure fraction exceeds 5% (MAX_FILL_FRAC = 0.05), the entire context is excluded from reporting to avoid artificial variance reduction.

IQR Screening and Hard Clipping: Outliers exceeding 3 times the interquartile range ( $\left[Q_{1}-3\text{IQR},Q_{3}+3\text{IQR}\right]$) are identified, and the evaluated output energy gaps are clipped to a maximum magnitude of ±50,000 aJ (± 5×10^−14^ J).

Variance Decomposition and Index Calculation: The output variance V(Δ*E*) is decomposed into main effects, pairwise interactions, and total-order effects:

(42) $$V\left(\text{Δ}E\right)=\sum_{i=1}^{D}V_{i}+\sum_{i<j}^{D}V_{ij}+\cdots +V_{1\ldots D}$$

First-Order Sensitivity Index ( $S_{1}^{\left(i\right)}$): Quantifies the fraction of total output variance attributable solely to parameter *θ_i_* acting individually:

(43) $$S_{1}^{\left(i\right)}=\frac{V_{i}}{V\left(\text{Δ}E\right)}=\frac{V_{\theta_{i}}\left(E_{\theta_{∼i}}\left[\text{Δ}E|\theta_{i}\right]\right)}{V\left(\text{Δ}E\right)}$$

Total-Order Sensitivity Index ( $S_{T}^{\left(i\right)}$): Quantifies the total contribution of parameter *θ_i_* to output variance, including all higher-order interactions with other parameters:

(44) $$S_{T}^{\left(i\right)}=\frac{E_{\theta_{∼i}}\left(V_{\theta_{i}}\left[\text{Δ}E|\theta_{∼i}\right]\right)}{V\left(\text{Δ}E\right)}$$

Second-Order Interaction Index ( $S_{2}^{\left(ij\right)}$): Measures the specific non-additive interaction variance between parameter pairs (*θ_i_*, *θ_j_*):

(45) $$S_{2}^{\left(ij\right)}=\frac{V_{ij}}{V\left(\text{Δ}E\right)}$$

Interaction Fraction (*f*_inter_): Measures the relative proportion of total variance driven by parameter interactions rather than additive main effects:

(46) $$f_{\text{inter}}=1-\frac{\sum_{i=1}^{D}S_{1}^{\left(i\right)}}{\sum_{i=1}^{D}S_{T}^{\left(i\right)}}$$

Confidence intervals (95%) for *S*_1_, *S_T_*, and *S*_2_ are calculated via bias-corrected bootstrap resampling with *N*_boot_ = 1000 resamples. Indices exceeding their corresponding 95% confidence bound ( $\left|S\right|>S_{\text{conf}}$) are designated as statistically resolved above background sampling noise. Together, the governing energy formulations, optimisation protocol and sensitivity indices used in this study are given in full in Equations 1–46.

## 3 Results

### 3.1 Rapid stabilization and robust numerical convergence of the energy optimization algorithm

Optimization using the L-BFGS-B algorithm demonstrated rapid and stable numerical convergence. The objective loss function Ɩ(*u*) dropped by nearly two orders of magnitude within the first 7 iterations, successfully stabilizing at a low minimum (7.5x 10^-3^) by iteration 20, confirming reliable parameter fitting without numerical instability (**Figure 1**).

**Figure 1:**
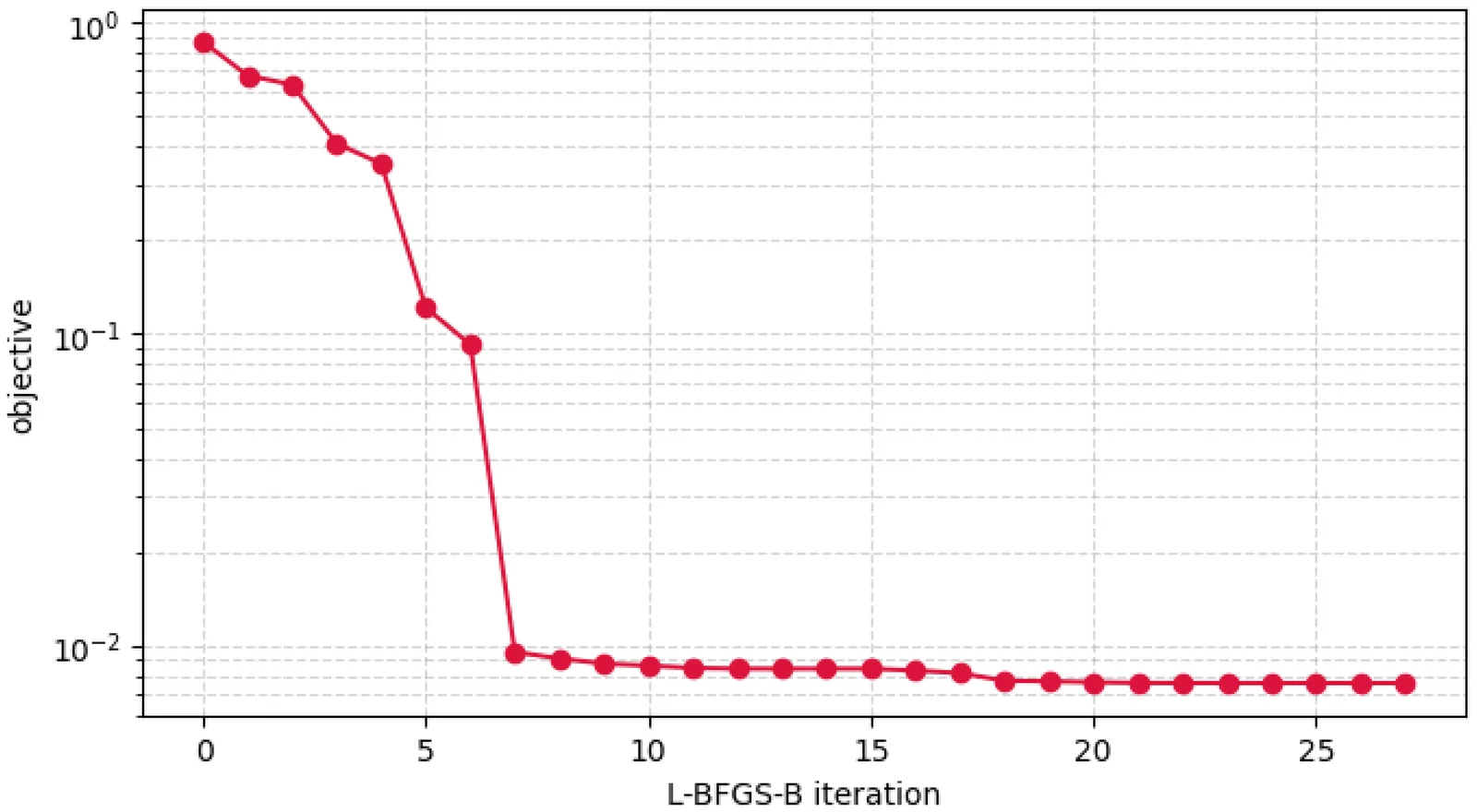
Convergence trajectory of the margin-maximizing loss function during L-BFGS-B optimization. Objective loss function L(u) plotted on a logarithmic scale (y-axis) across sequential optimizer iterations (x-axis). Red markers denote individual iteration steps, illustrating rapid loss stabilization at a minimum of 7.5 × 10^-^³ within 20 iterations.

### 3.2 Optimization reveals that flexible adhesion parameters adjust to energy margins while core mechanical properties remain stable.

During model parameterization, optimization trajectories across 27 L-BFGS-B iterations revealed distinct convergence behaviors among the free biophysical parameters while structural and reference properties were held fixed. Sensitive adhesion and signaling parameters underwent substantial adjustments from their initial states to satisfy directional energy margins—most notably “epsilon_ICAM” (converging to its upper bound of 2.0550×10^(-19) “ J”) and “K_CXCL10” (adjusting significantly to 6.3548×10^2 “ pM”)—whereas core mechanical parameters like “epsilon_PECAM1” (4.5610×10^(-20) “ J”), “DeltaG_burst” (7.0871×10^(-20) “ J”), and “DeltaG_SNARE” (1.6543×10^(-19) “ J”) remained highly stable throughout the iteration steps (**Figure 2**). Across all 16 optimized parameters, the L-BFGS-B algorithm effectively navigated the bounded search space to establish a uniquely identified parameter set that balances junctional unzipping, receptor-ligand kinetics, and membrane remodeling energies against fixed physiological constants such as baseline thermal energy (k_”B” T = 4.11×10^(-21) “ J”) and cellular dimensions (**Table 1**).

**Figure 2:**
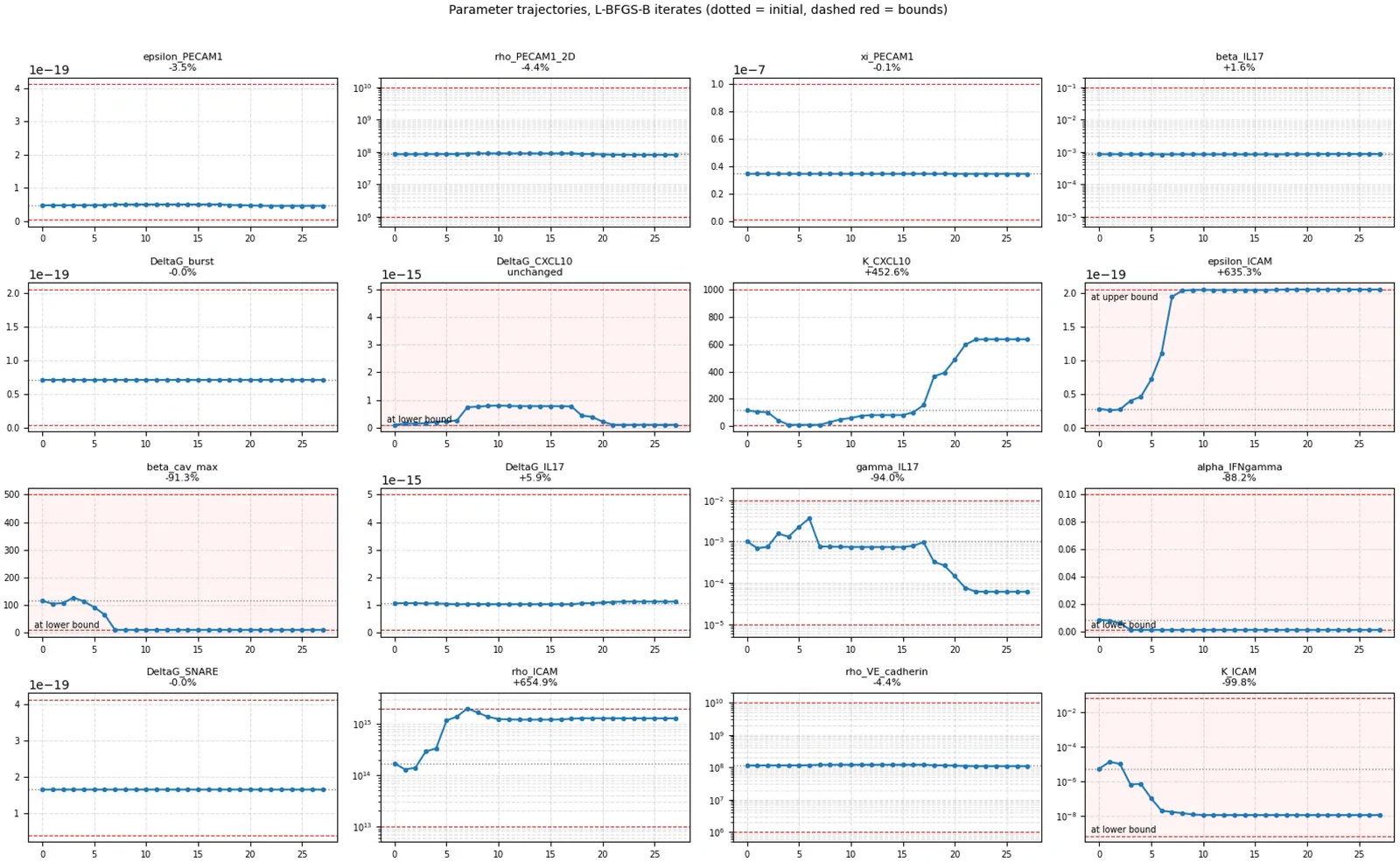
Convergence trajectories of unnormalized biophysical parameters. Trajectories of 16 free parameters across 27 L-BFGS-B iterations (x-axis) plotted against their physical values (y-axis). Initial parameter values are indicated by grey dotted lines, and allowable optimization bounds are marked by red dashed lines/shaded regions. Highly sensitive adhesion terms (ϵ_ICAM, K_CXCL10) adjust substantially to clear energy margins, whereas core mechanical parameters (ϵ_PECAM1, ΔG_burst, ΔG_SNARE) remain stable.

**Table 1:** Summary of parameters used in paracellular and transcellular migration.

Symbol	Physical meaning	Units	Energy term (route)	Range/bounds	Status	Value	Source	Classification
kT	Thermal energy	J	All terms	fixed	Fixed	4.11e-21	Physical constant	Constant
R_L	Leukocyte radius	m	E_L_deform, E_mem (both)	fixed	Fixed	4.50e-06	(4, 5)	Non-switching
h_cortex	Actin cortex thickness	m	E_cortex (trans)	fixed	Fixed	1.50e-07	(7, 8)	Structurally inert
V_cortex	Leukocyte cortical volume	m³	E_L_deform, E_Lc	derived	Fixed	3.82e-17	Derived from R_L, h_cortex	Derived
c_L	Leukocyte ground matrix stiffness	Pa	E_L_deform (parac)	fixed	Fixed	1.50e+01	Derived, E/6 (5)	Non-switching
k1_L	Leukocyte fibre stiffness	Pa	E_L_deform (parac)	fixed	Fixed	2.00e+01	(14); term inactive (I4<1)	Structurally inert
k2_L	Leukocyte fibre non-linearity	–	E_L_deform (parac)	fixed	Fixed	5.00e-01	(13) (model form); this study	Structurally inert
kf_L	Leukocyte fibre dispersion	–	E_L_deform (parac)	fixed	Fixed	1.00e-01	(15) (bounded 0-1/3)	Structurally inert
W_EC	Endothelial cell width	m	E_EC_retract (parac)	fixed	Fixed	1.00e-05	Derived from capillary lumen (16)	Non-switching
d_EC	Endothelial cell thickness	m	E_EC_retract, E_mem	fixed	Fixed	1.50e-06	(16, 17)	Switching (Mixed)
V_EC_ref	Endothelial reference volume	m³	E_EC_retract (parac)	fixed	Fixed	1.50e-16	Derived from W_EC, d_EC	Derived
c_EC	Endothelial ground matrix stiffness	Pa	E_EC_retract (parac)	fixed	Fixed	3.33e+02	Derived, E/6 (5)	Non-switching
k1_EC	Endothelial fibre stiffness	Pa	E_EC_retract (parac)	fixed	Fixed	8.69e+01	(13) (model form); this study	Switching (Th17)
k2_EC	Endothelial fibre non-linearity	–	E_EC_retract (parac)	fixed	Fixed	3.74e+00	(13) (model form); this study	Non-switching
kf_EC	Endothelial fibre dispersion	–	E_EC_retract (parac)	fixed	Fixed	1.00e-01	(15) (bounded 0-1/3)	Non-switching
L_junc	Junction contact length	m	E_unzip (parac)	fixed	Fixed	5.00e-06	(18)	Non-switching
g0	Resting junctional gap	m	g_eff, E_L_deform, E_EC_retract	fixed	Fixed	2.50e-06	(17, 18)	Switching (Th17)
epsilon_PECAM1	PECAM-1 bond energy	J	E_unzip (parac)	4.11e-21 to 4.11e-19	Free	4.56e-20	Fitted (this study)	Non-switching
rho_PECAM1_2D	PECAM-1 surface density	m^-^¹	E_unzip (parac)	1e6 to 1e10	Free	8.12e+07	Fitted (this study)	Non-switching
xi_PECAM1	PECAM-1 rupture reach	m	E_unzip (parac)	1e-9 to 1e-7	Free	3.45e-08	Fitted (this study)	Non-switching
epsilon_VE_cadherin	VE-cadherin bond energy	J	E_unzip (parac)	fixed	Fixed	2.47e-20	(19)	Non-switching
rho_VE_cadherin	VE-cadherin surface density	m^-^¹	E_unzip (parac)	1e6 to 1e10	Free	1.10e+08	Fitted (this study)	Non-switching
xi_VE_cadherin	VE-cadherin rupture reach	m	E_unzip (parac)	fixed	Fixed	3.00e-08	(19)	Non-switching
epsilon_claudin	Claudin bond energy	J	E_unzip (parac)	fixed	Fixed	2.47e-20	This study (set equal to VE-cadherin)	Non-switching
rho_claudin	Claudin surface density	m^-^¹	E_unzip (parac)	fixed	Fixed	1.00e+08	This study	Non-switching
xi_claudin	Claudin rupture reach	m	E_unzip (parac)	fixed	Fixed	3.00e-08	(20)	Non-switching
epsilon_occludin	Occludin bond energy	J	E_unzip (parac)	fixed	Fixed	2.47e-20	This study (set equal to VE-cadherin)	Non-switching
rho_occludin	Occludin surface density	m^-^¹	E_unzip (parac)	fixed	Fixed	1.00e+08	This study	Non-switching
xi_occludin	Occludin rupture reach	m	E_unzip (parac)	fixed	Fixed	3.00e-08	(20)	Non-switching
epsilon_JAM	JAM bond energy	J	E_unzip (parac)	fixed	Fixed	2.47e-20	This study (set equal to VE-cadherin)	Non-switching
rho_JAM	JAM surface density	m^-^¹	E_unzip (parac)	fixed	Fixed	1.00e+08	This study	Non-switching
xi_JAM	JAM rupture reach	m	E_unzip (parac)	fixed	Fixed	3.00e-08	This study	Non-switching
A_tip	Pseudopod tip area	m²	E_poly (both)	fixed	Fixed	7.90e-14	(21)	Structurally inert
delta	Actin monomer size	m	E_poly (both)	fixed	Fixed	2.70e-09	(21, 22)	Structurally inert
k_on	Actin association rate	M^-^¹ s^-^¹	E_poly (both)	fixed	Fixed	1.00e+06	(9)	Structurally inert
k_off	Actin dissociation rate	s^-^¹	E_poly (both)	fixed	Fixed	1.00e+00	(9)	Structurally inert
conc_A	Monomeric actin concentration	M	E_poly (both)	fixed	Fixed	1.00e-06	This study	Structurally inert
alpha_store	Actin storage fraction	–	E_poly (both)	fixed	Fixed	5.00e-01	This study	Structurally inert
N_fil	Filament number at tip	–	E_poly (both)	fixed	Fixed	8.80e+01	(21)	Structurally inert
DeltaG_IL17	IL-17 junction disruption energy	J	E_active (parac)	1e-16 to 5e-15	Free	1.13e-15	Fitted (this study)	Non-switching
K_IL17	IL-17 half-saturation	pg/mL	E_active (parac)	fixed	Fixed	1.05e+04	This study	Non-switching
beta_IL17	IL-17 sensitivity of PECAM-1 density	mL/pg	E_unzip (parac)	1e-5 to 0.1	Free	8.64e-04	Fitted (this study)	Non-switching
gamma_IL17	IL-17 dependence of gap opening	mL/pg	g_eff (parac)	1e-5 to 1e-2	Free	6.11e-05	Fitted (this study)	Non-switching
mu_L	Cytosolic shear modulus	Pa	E_Lc (trans)	fixed	Fixed	3.00e+01	(5)	Non-switching
sigma_cortex_L	Baseline cortical tension	Pa	E_cortex, E_gate (trans)	fixed	Fixed	3.00e+02	(5, 6)	Non-switching
delta_cl	Cortical layer thickness	m	E_cortex (trans)	fixed	Fixed	7.50e-08	(7, 8)	Non-switching
A_contact	Leukocyte contact area	m²	E_cortex, E_ICAM (trans)	fixed	Fixed	6.36e-11	Derived from R_L	Switching (Mixed)
h_mem	Membrane deformation depth	m	E_mem (trans)	fixed	Fixed	1.00e-08	This study	Non-switching
kappa_b	Membrane bending modulus	J	E_mem (trans)	fixed	Fixed	9.25e-20	(10)	Non-switching
gamma_mem	Baseline membrane tension	N/m	E_mem (trans)	fixed	Fixed	2.00e-03	(12, 23)	Switching (Th17, Mixed)
n_cav_max	Available caveolar pool	–	E_fus (trans)	fixed	Fixed	1.30e+01	Derived from d_EC, r_cav	Non-switching
n_star	Fusion events before burst	–	E_fus (trans)	fixed	Fixed	6.00e+00	This study	Non-switching
DeltaG_SNARE	SNARE fusion free energy	J	E_fus (trans)	4.11e-20 to 4.11e-19	Free	1.65e-19	Fitted (this study)	Non-switching
DeltaG_hemi	Hemifusion free energy	J	E_burst (trans)	fixed	Fixed	2.88e-19	(11)	Non-switching
DeltaG_burst	Pore burst free energy	J	E_burst (trans)	4.11e-21 to 2.06e-19	Free	7.09e-20	Fitted (this study)	Non-switching
alpha_IFNgamma	IFN-γ sensitivity of Cav-1 induction	mL/pg	Cytokine coupling, E_mem (trans)	1e-3 to 0.1	Free	1.00e-03	Fitted (this study)	Non-switching
fold_cap	Ceiling on Cav-1 induction	–	Cytokine coupling (trans)	fixed	Fixed	5.00e+00	This study	Non-switching
beta_cav_max	Maximum caveolar reserve amplification	–	Cytokine coupling, E_mem (trans)	10 to 500	Free	1.00e+01	Fitted (this study)	Non-switching
rho_ICAM	ICAM-1 surface density	m^-^²	E_ICAM, caveolar gating (trans)	1e13 to 2e15	Free	1.28e+15	Fitted (this study)	Switching (Th1, Mixed, PathTh17)
epsilon_ICAM	ICAM-1 bond energy	J	E_ICAM (trans)	4.11e-21 to 2.06e-19	Free	2.06e-19	Fitted (this study)	Switching (Th1, Mixed, PathTh17)
K_ICAM	ICAM-1 engagement half-saturation	J m^-^²	Caveolar gating (trans)	6.58e-10 to 6.58e-2	Free	1.16e-08	Fitted (this study)	Switching (Th1, Mixed, PathTh17)
DeltaG_CXCL10	CXCL10 guidance free energy	J	E_CXCL10 (trans), cortical stiffening (parac)	1e-16 to 5e-15	Free	1.00e-16	Fitted (this study)	Non-switching
K_CXCL10	CXCL10 half-saturation	pg/mL	E_CXCL10 (trans), cortical stiffening (parac)	10 to 1000	Free	6.35e+02	Fitted (this study)	Non-switching
stiff_max	Maximum CXCL10 cortical stiffening	–	E_L_deform (parac)	fixed	Fixed	1.00e+01	This study	Non-switching
CXCL10_ICAM_enhancement _factor	CXCL10 enhancement of ICAM-1 engagement	–	E_ICAM (trans)	fixed	Fixed	1.00e+01	This study	Structurally inert

### 3.3 Biphasic route regulation: high IFN-γ dominance suppresses moderate IL-17 elevations prior to a steep paracellular switch

In case, of Baseline Route Commitment (Low to Moderate IL-17 and In a high IFN-γ background (IFN-γ = 1000 pg/mL), the cell strongly commits to the transcellular route (s = −1.0) across all IL-17 concentrations up to approximately 1000 pg/mL. At the baseline Th1 condition (IL-17 = 10 pg/mL), the median trajectory and all feasible parameter sets remain around s ≈ −1.0. In cases of moderate IL-17 elevation, these increases are insufficient on their own to overcome the strong transcellular drive established by IFN-γ and CXCL10. Therefore, the transcellular pathway remains energetically favorable across a broad physiological window. A steep, non-linear transition occurs as the IL-17 concentration approaches higher levels. During this phase, the route preference metric rapidly crosses the energetic parity threshold (s = 0, red dashed line) and shifts into the paracellular regime (**Figure 3**).

**Figure 3:**
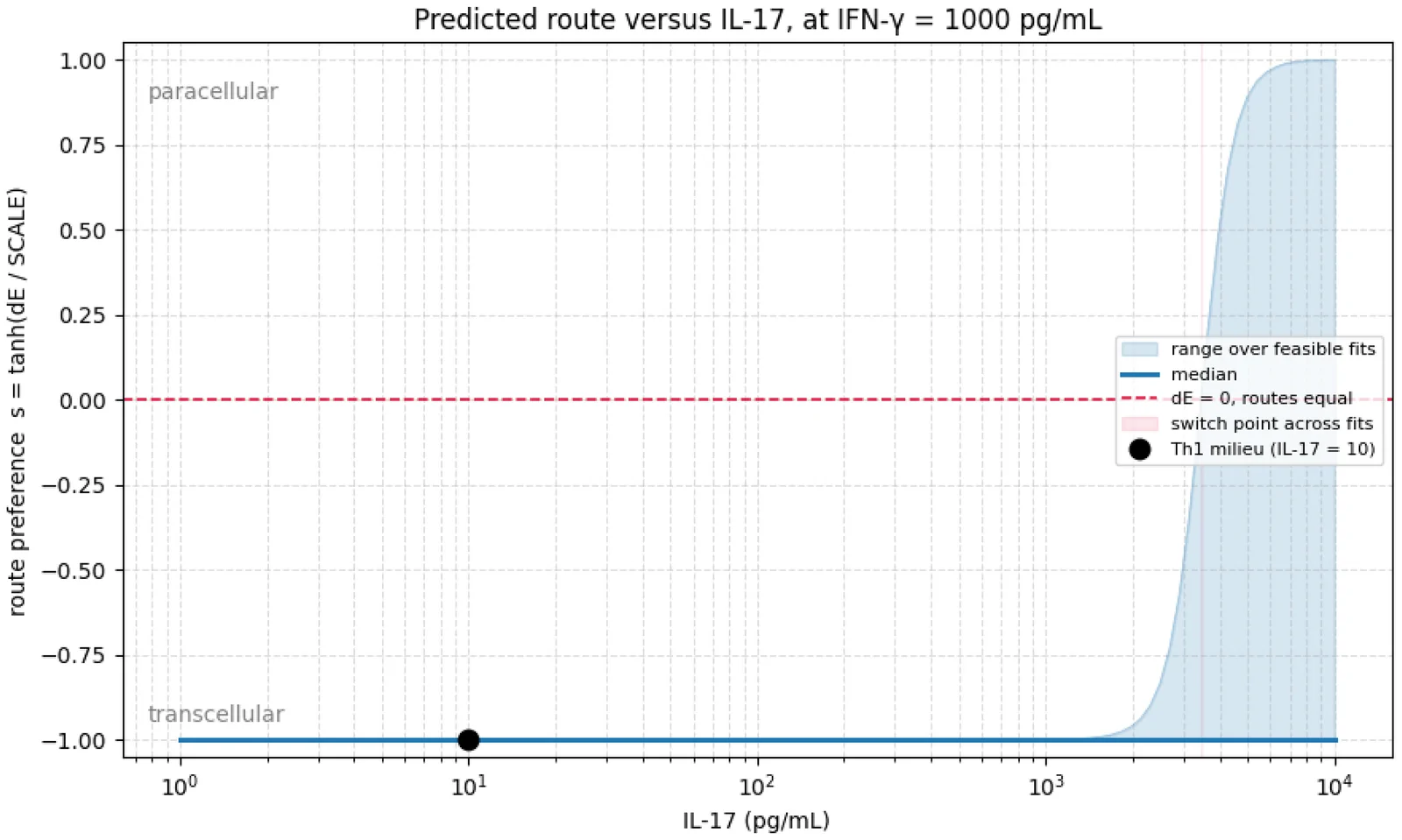
Biphasic route switching across an IL-17 gradient under high IFN-γ background. Predicted transmigration route preference metric s = tanh(ΔE/E_scale) (y-axis, where s = −1 represents total transcellular preference, s = 0 indicates thermodynamic parity, and s = +1 represents total paracellular preference) plotted against logarithmic IL-17 concentration (x-axis, pg/mL) under fixed Th1 background conditions (IFN-γ = 1000 pg/mL, CXCL10 = 500 pg/mL). The solid blue line tracks median ensemble predictions, the light blue band shows fit uncertainties, the black dot indicates baseline Th1 milieu (IL-17 = 10 pg/mL), and the pink shaded band marks crossover points (ΔE = 0) across multi-start fits.

### 3.4 IFN-*γ* dominance predict a transcellular route, while a rise in IL17 seem to be associated with paracellular route

Our findings shows that route selection across the 2D cytokine plane is primarily governed by the vertical IFN-*γ* axis. High IFN-*γ* levels ( ≳250 pg/mL) strongly favor transcellular migration (*s* → −1.0, red region), whereas low IFN-*γ* levels ( ⪅200 pg/mL) favor paracellular migration (*s* → +1.0, blue region). The sharp black contour at IFN-γ≈230 pg/mL designates the thermodynamic switching threshold where Δ*E* = 0 (**Figure 4**). Interestingly, minor parameter dependence (*A_ij_* < 0.8, green/purple bands) is restricted to a narrow horizontal band surrounding the decision boundary ( IFN-γ≈100--300 pg/mL). This demonstrates that parameter variations only shift the precise threshold position slightly without disrupting the model’s global prediction topology (**Figure 4**).

**Figure 4:**
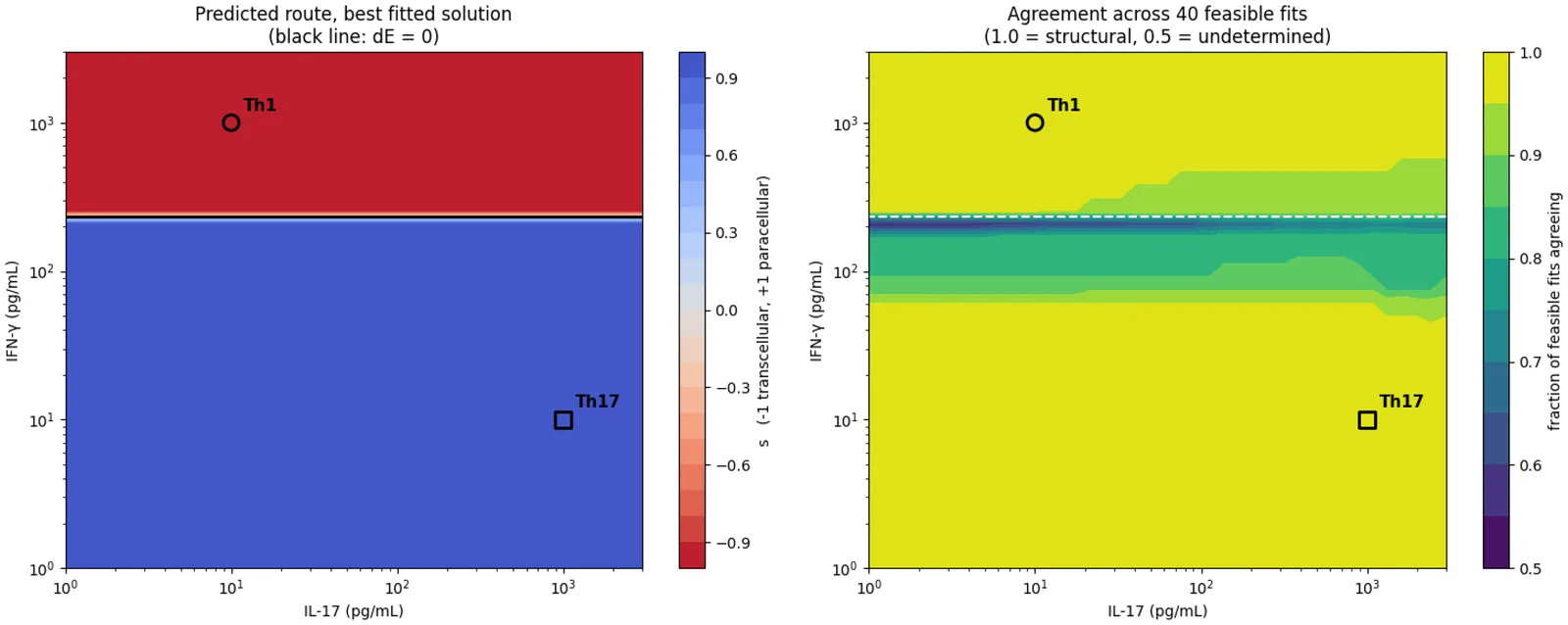
Two-dimensional cytokine phase landscape and prediction consensus. (Left) Directional pathway preference metric s plotted across continuous logarithmic axes of IL-17 (x-axis, pg/mL) and IFN-γ (y-axis, pg/mL). Red regions (s< 0) indicate transcellular preference, blue regions (s > 0) indicate paracellular preference, and the black line marks thermodynamic parity (ΔE = 0). Annotated points highlight reference Th1 (circle) and Th17 (square) states. (Right) Structural agreement metric A_ij across N = 40 feasible multi-start fits (y-axis: IFN-γ; x-axis: IL-17). Bright yellow indicates 100% prediction consensus (A_ij = 1.0) across all fits.

### 3.5 Transition into inflammatory conditions shifts cells preferences

Global variance-based sensitivity analysis reveals a fundamental shift in the mechanical control architecture governing transmigration route selection ( $\text{Δ}E=E_{\text{trans}}-E_{\text{parac}}$) as cells transition between inflammatory microenvironments. In conditions dominated by IFN-γ (Th1, Mixed, and PathTh17 contexts), route preference variance is heavily dictated by ICAM-1 adhesion kinetics (*K*_ICAM_ and *ρ*_ICAM_) acting via direct main effects, with total sensitivity indices reaching $S_{T}\approx 0.57\text{--}0.59$. Conversely, in an IL-17–dominant milieu (Th17 context), ICAM-1 sensitivity vanishes completely (*S_T_* = 0.00), and control shifts entirely to PECAM-1 physical properties (*ρ*_PECAM1_2D_, *S_T_* = 0.80) alongside IL-17 signaling parameters (*β*_IL17_ and *γ*_IL17_). Crucially, while IFN-γ-containing environments operate via largely independent parameter contributions (where direct main effects *S*_1_ account for roughly 64—66% of total variance), the pure Th17 context is strongly non-additive, with high-order parameter interactions driving 76% of all switching variance. Specific second-order pairwise interaction mapping resolves these non-linearities predominantly to coupling between PECAM-1 spatial density (*ρ*_PECAM1_2D_) and its mechanical energy scaling factor ( $\in_{PECAM1}$) during pure Th17 stimulation, whereas co-stimulation environments exhibit subtle cross-talk between ICAM-1 binding, VE-cadherin density, and cytokine responsiveness. Across all contexts, non-regulatory base thermodynamic scale parameters (Δ*G*_burst_, Δ*G*_SNARE_, Δ*G*_CXCL10_, Δ*G*_IL17_) remain consistently below statistical noise bounds (*S_T_* < 0.01), confirming that transmigration route selection is controlled by specific adhesion-receptor expression and signaling dynamics rather than basal energetic offsets (**Figure 5**).

**Figure 5:**
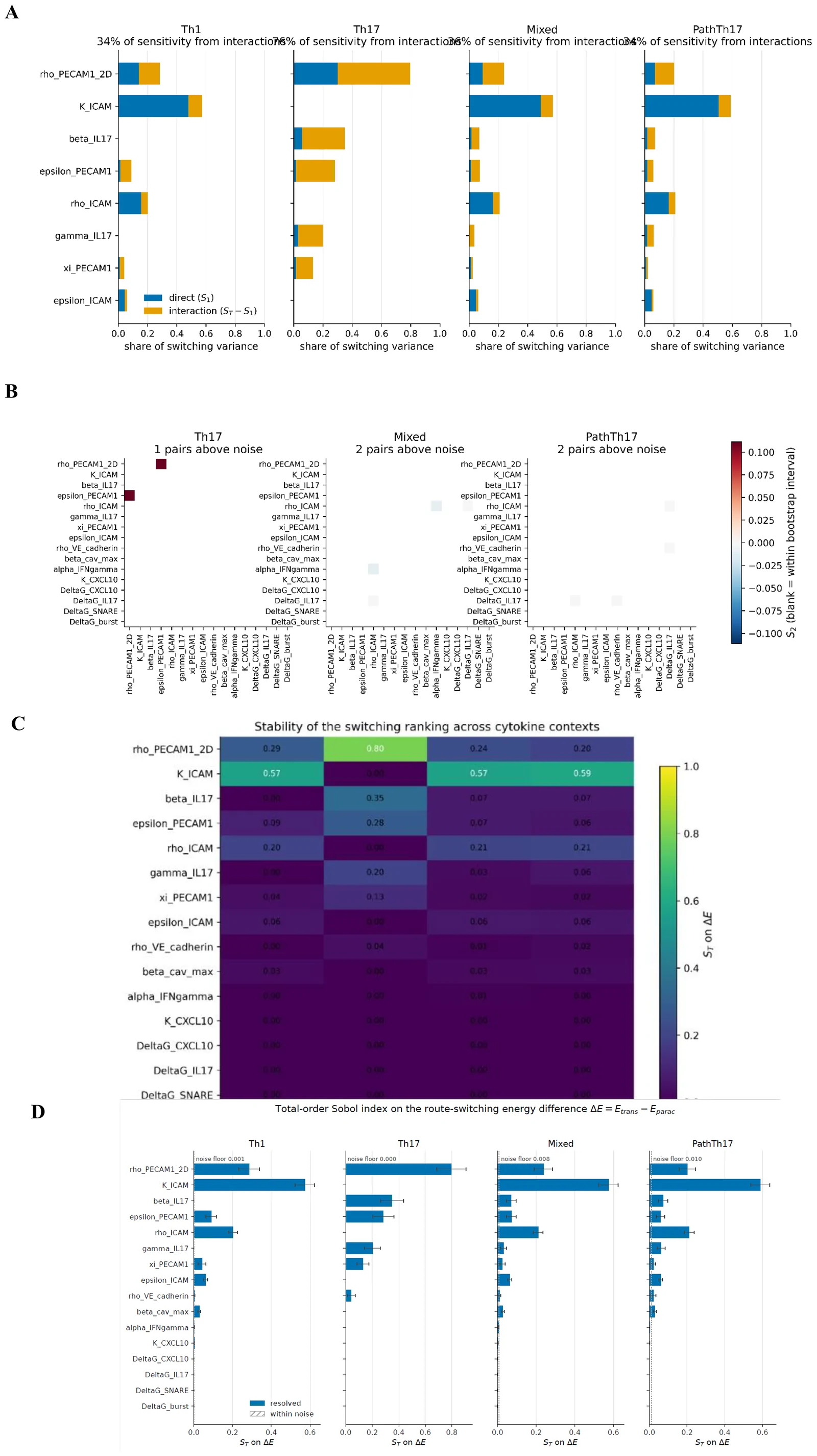
Global Sobol sensitivity decomposition across distinct inflammatory environments. **(A)** Decomposition of total switching energy variance (ΔE = Etrans − Eparac) into direct main effects (S1, blue) and higher-order parameter interactions (ST − S1, gold) for key parameters across four cytokine contexts (x-axis: variance share; y-axis: parameters). **(B)** Total-order Sobol indices (ST, x-axis) with 95% bootstrap confidence intervals for evaluated parameters (y-axis). Solid blue bars indicate indices resolved above context noise floors (dashed vertical lines). **(C)** Parameter Stability Heatmap: context-dependent matrix of total-order sensitivity indices (ST) across evaluated parameters. Colour intensity ranges from purple (ST ≈ 0, no sensitivity) to bright green (ST ≈ 1, dominant control), highlighting the complete divergence in control architecture between ICAM-1-driven (Th1/Mixed/PathTh17) and PECAM-1-driven (Th17) signalling regimes. **(D)** Pairwise Interaction Matrices: second-order interaction indices (S2) resolving specific pairwise parameter couplings above bootstrap noise. Dark red entries indicate strong non-additive pairwise interactions (predominantly in pure Th17), whereas light blue/white entries indicate weak or negligible pairwise coupling. Blank entries fall within the bootstrap noise interval.

## 4 Discussion

In this study, we simulate the decision pathway of Th1 and Th17 cells infiltrating the blood-brain barrier. We built an optimizer using the L-BFGS-B technique and validated its results using the Trust Region Reflective (TRF) technique. Our optimizer is built on the hypothesis that for cells to carry out a process efficiently, they minimize the total energy they expend. Thus, rather than following a predetermined biological program, cells use available physical mechanisms to infiltrate the blood-brain barrier. This results in the leukocyte’s choice between paracellular and transcellular passage being governed by environmental switches. These switches are primarily driven by the cytokine axis. The interaction between IFN-γ, IL-17, and other downstream chemokines leads to the regulation of specific receptors that support one pathway over the other. Specifically, IFN-γ-driven signaling couples with ICAM-1 and Cav-1 kinetics (25) to support the transcellular route. Conversely, IL-17-mediated signaling actively loosens junctions and relies on PECAM-1 physical properties to favor paracellular transit. This architecture uncovers a dramatic shift in global mechanical control, moving from ICAM-1 dominance to PECAM-1 and non-additive interactions as the inflamed microenvironment transitions from Th1- to Th17-dominant neuroinflammation.

The foundational premise of our model—that leukocytes minimize energetic expenditure to govern route selection—aligns tightly with established thermodynamic theories of cellular behavior. Previous migration studies heavily support this physical “path of least resistance” strategy (1, 24). For instance, Lomakin et al. reported that cells actively employ their nucleus as an intracellular ruler to sense physical confinement, dynamically modulating actomyosin contractility to navigate restrictive spaces based on energetic optimization (2) Similarly, Renkawitz et al. demonstrated that fast-moving immune cells position their stiff nucleus at the front of the cell body to act as a mechanical gauge, explicitly steering the leukocyte along the pathway that minimizes mechanical resistance and nuclear deformation costs. Furthermore. It was suggested that, T cells, during diapedesis, act to minimize thermodynamic energy by locating the weakest spot to penetrate the blood-brain barrier. This process reduces the ATP required for actin polymerization and avoids steep energy penalties from membra strain (1). However, Wolf et al. and Friedl et al. reported that cells do not always passively choose pre-existing low-energy paths; instead, complex phenotypes can deploy a slow, path-generating strategy using pericellular proteolysis, actively cutting through dense extracellular boundaries when the energetic cost of physical squeezing becomes entirely prohibitive (3). By formalizing this balance computations across both the leukocyte and the yielding endothelium, our framework extends these localized mechanical observations into a unified, predictive cytokine-driven energy landscape.

Our result indicate that a complex interplay between various cytokines produced by the cell determine the pathway used. In case of a high IFN-γ background (1000 pg/mL), the cell strongly commits to the transcellular route (*s* = −1.0) across all IL-17 concentrations up to ∼1000 pg/mL. Resistance to Cytokine Flipping: moderate elevations in IL-17 are insufficient on their own to overcome the strong transcellular drive established by IFN-γ and CXCL10. Non-Linear Pathway Switching: a steep, non-linear transition to the paracellular regime (*s* → +1.0) occurs only when IL-17 concentration approaches extreme supraphysiological levels (∼2000–5000 pg/mL). The reason behind this is that route preference variance is heavily dictated by ICAM-1 adhesion kinetics (*K_ICAM_* and *ρ_ICAM_*) acting via direct main effects, with total sensitivity indices reaching *S_T_* ≈ 0.57–0.59. These contexts operate via largely independent parameter contributions, where direct main effects (*S*_1_) account for 64–66% of total variance. When IL-17 sensitivity values are high, the mechanical control shifts entirely to PECAM-1 physical properties (*ρ_PECAM_*_1_2_*_D_*, *S_T_* = 0.80) alongside IL-17 signaling parameters (*β_IL_*_17_ and *γ_IL_*_17_). Non-Additive High-Order Interactions: the pure Th17 context is strongly non-additive, with high-order parameter interactions driving 76% of all switching variance. Specific second-order pairwise interaction mapping resolves these non-linearities predominantly to the coupling between PECAM-1 spatial density (*ρ_PECAM_*_1_2_*_D_*) and its mechanical energy scaling factor ( $\in_{PECAM1}$). In agreement with previous reports, our model predicts that knocking out Cav-1 flips the migration pathway into the paracellular route. This indicates that Cav-1 serves as the crucial downstream mechanical reservoir that physically enables the transcellular route. While ICAM-1 and IFN-γ act as the upstream biochemical switches, Cav-1 provides the actual physical infrastructure that lowers the energetic cost of forming a transcellular pore. This demonstrates that biochemical signaling alone is not sufficient to guarantee transcellular migration; instead, successful pore formation requires the simultaneous availability of a permissive mechanical scaffold provided by Cav-1-dependent membrane organization. Taken together, these findings reveal a hierarchical, two-tiered gating architecture where upstream biochemical signals dictate the pathway command, but downstream physical reservoirs strictly govern structural execution.

While our thermodynamic framework provides robust and structurally invariant predictions, several inherent limitations must be acknowledged. Fixed Geometric and Physical Parameters. First, baseline physical dimensions and certain mechanical constants were held fixed or estimated based on established values in the available biophysical literature. This constraints-based approach was deliberately chosen to mitigate parameter unidentifiability issues common to high-dimensional biological systems. Given the large number of parameters, unconstrained multi-variable optimization risks stalling in non-unique local minima. By locking well-characterized cell geometries, we restricted the model’s free degrees of freedom primarily to signaling and receptor-ligand kinetics. Because cytokine concentrations are highly intuitive variables that are easily manipulated *in vitro* and *in vivo*, focusing the optimization on these parameters ensures that our dose-response thresholds and 2D phase maps are readily testable and experimentally verifiable. Omission of Proteolytic Degradation. Second, our framework currently excludes parallel active pathways that leukocytes utilize to clear physical cellular barriers. Specifically, we did not incorporate the focal release of matrix metalloproteinases, such as MMP-9. This enzyme actively cleaves and disintegrates junctional adhesion molecules to loosen endothelial barriers—a mechanism extensively documented in both inflammatory leukocyte trafficking and metastatic cancer cell migration. Future iterations of this framework will aim to couple hyperelastic cell deformation with kinetic models of localized enzymatic digestion.

## 5 Conclusion

Overall, this report represents a novel attempt to model a highly complex, multi-cellular sorting system based entirely on the principle of minimal energy optimization. Our optimization protocol and sensitivity maps strongly indicate that transmigration route selection is not a predetermined, hardwired program. Instead, the final pathway choice emerges as a dynamic outcome driven by intricate, context-dependent parameter interactions. It is a real-time behavioral switch controlled entirely by the local biochemical microenvironment and the specific mechanical constraints available to the cells at that precise point in time and space.

## Data Availability

The original contributions presented in the study are included in the article/supplementary material. Further inquiries can be directed to the corresponding authors.
